# Hematobiochemical profile in Surti goats during post-partum period

**DOI:** 10.14202/vetworld.2016.19-24

**Published:** 2016-01-12

**Authors:** Tanvi D. Manat, Sandhya S. Chaudhary, Virendra Kumar Singh, Sanjay B. Patel, Gopal Puri

**Affiliations:** Department of Veterinary Physiology and Biochemistry, College of Veterinary Sciences and Animal Husbandry, Navsari Agricultural University, Navsari - 396 450, Gujarat, India

**Keywords:** hematobiochemical parameters, post-partum period, Surti goat

## Abstract

**Aim:**

The study was undertaken to find out the changes in hematobiochemical profile in post-partum Surti goats.

**Materials and Methods:**

The present study was conducted on 40 Surti goats out of which 20 goats who had undergone recent parturition acted as treatment group and 20 non-pregnant animals comprised control group. Blood samples were collected from the treatment group on 0, 7, 14, 21, 30, and 45 days post kidding and once from the control group. Blood samples were analyzed for hematological parameters such as hemoglobin (Hb), packed cell volume (PCV), total erythrocyte count (TEC), total leukocyte count (TLC), differential leukocyte count, and biochemical metabolites such as total protein (TP), albumin, globulin, total cholesterol (TC), triglycerides (TG), non-esterified fatty acid (NEFA), glucose, and urea.

**Results:**

Hb level was the highest on the 45^th^ day and lowest on the day of parturition. Significantly low level of Hb, PCV, and lymphocyte was found on 0 day and significantly high level was found on the 45^th^ day. TEC was also low on the 0 day and high on the 45^th^ day post kidding, but the difference was non-significant. Mid-sized cells and granulocyte percentage decreased significantly from 0 to 45^th^ day post-partum. TP, albumin, and urea were found to be lowest on 0 day and highest on 45^th^ day post-partum. Urea concentration increased parallel to TP indicating an increase in urea production with catabolism of protein. The globulin concentration also increased from 0 to 45^th^ day post-partum, but the difference was non-significant. TC, TG, and NEFA were the highest on 0 day and lowest on the 45^th^ day post-partum. Decrease in TC and TG from 0 to 45^th^ day post-partum indicates that the animals utilized the lipids for the supply of energy for milk production. High level of NEFA acts as an indicator of negative energy status but in the present study, the high glucose level on 0 day indicates that the animals were in positive energy status. The glucose concentration decreased up to 14^th^ day post-partum and then started increasing from 21^st^ day onward.

**Conclusion:**

Hematobiochemical parameters are indicative of health status of animals. Significantly low values of Hb, PCV, and TLC during first 2 weeks post-partum are indicative of stress. Catabolism of protein occurred during 2 weeks post-partum, as observed from increase in urea concentration. Decrease TGs and high level of NEFA during the transition period are suggestive of utilization of lipids for the supply of energy. High glucose level on 0 day indicates that the animals were in positive energy status.

## Introduction

India comprises of 135.17 million goat population out of which 3.67% belongs to Gujarat as per 19^th^ Livestock Census of the year 2012. Surti goat is a medium sized mostly stall-fed, non-nomadic in nature, dual purpose goat breed and found in small towns and cities. The breed is confined to the middle and south region of Gujarat state and neighboring areas of Maharashtra state up to Nasik district. Surti goats are famous for their fertility, prolificacy, meat and milk quality as well as adaptability to the hot humid condition. Goats have unique ability to adapt and maintain themselves in harsh environments and elicit great variation in hematological and biochemical parameters on the basis of the physiological phase of life even within the same breed. It has distinct social, economical, managerial, and biological advantages over other livestock species and often termed as the “poor man’s cow.”

Hematological parameters are good indicators of physiological health status, and its evaluation is important in assessing the response of animal to various physiological stressful conditions such as pregnancy, parturition, and lactation. Metabolites *viz*. total protein (TP), triglycerides (TGs), free fatty acids, and urea are important indicators of the health and nutritional status of the animals [1] and metabolic activity in lactating animals [2]. The post-parturient period is characterized by marked changes in an animal’s endocrine and metabolic status as well as by reduction in feed intake when the nutrient demand for impending lactogenesis is increasing.

In spite of its good production potential, the Surti breed has been declared as an endangered breed since 80’s owing to its possibilities of extinction, but very little efforts are made toward its conservation as gazed by very few studies done on this precious goat breed of South Gujarat. Post-partum period, a part of the transition period, especially upto 45 days after parturition is the most stressful period because of the depressed feed intake and endocrine and metabolic changes at parturition and lactation. Optimal transition requires a comprehensive understanding of the hematobiochemical events occurring during the periparturient period. Breed to breed variation in hematobiochemical parameters during the transition period also occurs within the same species of the animal because of the climatic condition of the region of which animals are inhabitants.

The present study was therefore planned to get detailed information through frequently sampling on routinely measured hematological and biochemical analytes during the post-parturient period in Surti goats.

## Materials and Methods

### Ethical approval

The study was conducted following approved guidelines of the Institutional Animal Ethics Committee.

### Location of study and climatic conditions

The present study was conducted in the Department of Veterinary Physiology and Biochemistry. The experimental animals were maintained at Livestock Research Station, Navsari Agricultural University, Navsari, which is geographically located approximately at an altitude of 11.89 m above mean sea level, at latitude of 20°-57’0’’ North and longitude of 72°-54’0’’ East. The climate of the area forms the part of the tropical and coastal area. In general, winter is cool and dry while summer and monsoon remain hot and humid. During the study period, temperature ranged from 31.4°C (maximum) to 17.5°C (minimum) and relative humidity ranged from 96.7% (maximum) to 39.7% (minimum).

### Experimental animals

40 apparently healthy Surti goats (aged 36-51 months) were selected for the study and divided into two groups (treatment and control) of 20 each. All the goats were housed in pucca shed with concrete floor, and feeding was done as per the ICAR feeding standards, 1998. The duration of study in the treatment group was 45 days post-partum wherein blood was collected on the day of kidding, 7^th^, 14^th^, 21^st^, 30^th^, and 45^th^ days post-partum. Blood was also collected once from control group which comprised of non-pregnant goats. Parity of all the goats ranged from first to sixth. Approximately 5 ml of whole blood from each animal was collected from a jugular vein in K_3_ ethylenediaminetetraacetic acid vacutainers for hematological examination. Serum from clotted blood was separated by centrifugation at 3000 rpm for 15 min and stored at −20°C in deep freeze until analyzed for biochemical parameters.

### Hematological analysis

Hematological parameters such as ­hemoglobin (Hb), packed cell volume (PCV), total erythrocyte count (TEC), total leukocyte count (TLC), and differential leukocyte count were estimated by fully automated hematology cell counter (MEDONIC CA 620/530 VET).

### Biochemical analysis

The biochemical metabolites were analyzed by the use of Randox kits on semi-automated clinical chemistry analyzer (Merck). The serum biochemical metabolites measured were TP (g/dl) by biuret method, albumin (g/dl) by bromocresol green dye binding method, globulin (g/dl), total cholesterol (TC) (mg/dl) by enzymatic endpoint method, TG (mg/dl) by glycerol-3-phosphate oxidase-trinder method, non-esterified fatty acid (NEFA) (mmol/L) by colorimetric method, glucose (mg/dl) by GOD-PAP method, and urea (mg/dl) by glutamate dehydrogenase method.

### Statistical analysis

The collected data for all the parameters were analyzed using randomized block design [3].

## Results

The values of different hematological parameters during the post-partum period on 0, 7^th^, 14^th^, 21^st^, 30^th^, and 45^th^ days are given in Table-1. The analysis of variance (ANOVA) for different hematological parameters is presented in Table-2.

**Table-1:** Hematological profile (mean±SE) in Surti goats during the post-partum period.

Parameters	0 day	7 day	14 day	21 day	30 day	45 day	Control	Reference values
Hb (g/dl)	6.10±0.13^a^	7.32±0.07^b^	8.09±0.09^c^	8.52±0.07^d^	9.16±0.06^e^	9.54±0.08^f^	10.18±0.22^h^	8-12 [22]
PCV (%)	17.13±0.29^a^	22.66±0.53^b^	23.90±0.47^c^	24.02±0.34^c^	24.49±0.36^c^	24.82±0.42^c^	26.64±0.32^d^	22-38 [22]
TEC (×10^6^/mm^3^)	13.10±0.43	13.13±0.57	13.27±0.55	13.49±0.81	13.71±0.56	13.99±0.48	14.12±0.27	8-18 [22]
TLC (×10^3^/mm^3^)	10.56±0.43^a^	12.04±0.52^ab^	12.99±0.63^b^	12.75±0.54^b^	12.47±0.65^b^	12.26±0.57^b^	12.31±0.32^b^	4-13 [22]
LYM (%)	51.80±0.36^a^	55.65±0.57^b^	59.00±0.55^c^	61.95±0.46^d^	64.50±0.35^e^	66.85±0.25^f^	51.95±0.39^a^	50-70 [22]
MID (%)	5.90±0.24^e^	5.80±0.22^e^	4.80±0.16^c^	4.05±0.18^b^	3.90±0.18^ab^	3.50±0.11^a^	4.95±0.25^cd^	0-4 [22]
GRAN (%)	42.30±0.42^f^	38.55±0.58^e^	36.20±0.51^d^	33.90±0.54^c^	31.70±0.39^b^	29.65±0.27^a^	32.40±0.43^b^	30-48 [22]

Mean bearing different superscript differ significantly. Hb=Hemoglobin, PCV=Packed cell volume, TEC=Total erythrocyte count, TLC=Total leukocyte count, SE=Standard error

**Table-2:** Analysis of variance for hematological parameters.

Source of variation	df	Mean sum of square						

		Hb	HCT	TEC	TLC	LYM	MID	GRAN
Treatment	6	38.65**	180.45**	3.35	12.43*	710.33**	17.42**	382.67**
Error	133	0.27	3.16	5.99	5.74	3.72	0.77	4.22

* p<0.05,

** p<0.01.

Hb=Hemoglobin, PCV=Packed cell volume, TEC=Total erythrocyte count, TLC=Total leukocyte count

Hb level was significantly (p<0.01) different between the days and groups. The highest and lowest concentration of Hb was found on the 45^th^ day post-partum and on the day of kidding, respectively. PCV values increased significantly (p<0.05) from 0 day to 14^th^ day. The TEC did not show significant differences during the post-partum period. Significantly (p<0.01) low value of TLC was observed on 0 day but the difference was non-significant between the groups and from 7^th^ to 45^th^ day post-partum. Lymphocyte % increased from the lowest value on 0 day to highest value on 45^th^ day post-partum and showed a significant (p<0.01) difference between treatment and control group. However, the descending trend was observed for the MID% and granulocyte % from 0 to 45^th^ day post-partum.

The values for all hematological parameters in both the groups were in normal range except GRAN% which was slightly lower on 45^th^ day post-partum.

The values of different biochemical parameters *viz*. TP, albumin, globulin, TC, TG, glucose, NEFA, and urea during the post-partum period are presented in Table-3. The ANOVA for different biochemical parameters is presented in Table-4.

**Table-3:** Biochemical metabolites (mean±SE) in blood serum/plasma of Surti goats.

Parameters	0 day	7 day	14 day	21 day	30 day	45 day	Control	Reference values
TP (g/dl)	5.35±0.12^a^	6.16±0.03^b^	6.77±0.10^c^	7.11±0.14^de^	7.19±0.17^e^	7.36±0.11^e^	6.84±0.13^cd^	6.9±4.8 [23]
Albumin (g/dl)	2.49±0.10^a^	3.17±0.08^b^	3.52±0.08^c^	3.77±0.12^cd^	3.81±0.08^d^	3.92±0.14^d^	3.74±0.10^cd^	3.3±0.33 [23]
Globulin (g/dl)	2.86±0.16	2.99±0.08	3.25±0.16	3.34±0.22	3.39±0.18	3.44±0.19	3.11±0.20	3.6±0.5 [23]
TC (mg/dl)	83.95±1.47^f^	71.45±0.71^d^	60.20±1.24^c^	57.00±0.66^b^	55.45±0.11^ab^	52.90±0.28^a^	75.10±1.12^e^	80±130 [23]
TG (mg/dl)	49.76±1.00^d^	43.41±0.74^c^	38.76±1.08^b^	37.39±0.80^ab^	36.08±0.82^a^	35.98±0.67^a^	36.98±0.75^ab^	8.85-26.55 [24]
NEFA (mmol/L)	0.39±0.01^e^	0.37±0.01^de^	0.35±0.01^d^	0.26±0.01^c^	0.17±0.02^b^	0.13±0.01^a^	0.50±0.01^f^	0.20-0.21 [18]
Glucose (mg/dl)	84.49±1.96^e^	62.49±0.77^a^	63.55±0.64^a^	71.51±0.75^bc^	74.51±1.28^cd^	76.58±1.77^d^	69.07±0.95^b^	62.8±7.1 [23]
Urea (mg/dl)	19.66±1.83^a^	24.29±1.54^b^	30.37±1.39^c^	34.71±1.44^d^	39.68±1.78^e^	43.40±1.97^e^	31.54±0.50^cd^	33.73±4.27 [23]

Mean bearing different superscript differ significantly. TP=Total protein, TC=Total cholesterol, TG=Triglycerides, NEFA=Non-esterified fatty acid, SE=Standard error

**Table-4:** Analysis of variance for biochemical metabolites.

Source of variation	df	Mean sum of square							

		TP	Albumin	Globulin	TC	TG	NEFA	Glucose	Urea
Treatment	6	9.99**	5.09**	0.95	2757.37**	518.48**	0.34**	1177.74**	1369.92**
Error	133	0.29	0.21	0.63	17.13	14.36	0.00	31.73	48.56

** p<0.01.

TP=Total protein, TC=Total cholesterol, TG=Triglycerides, NEFA=Nonesterified fatty acid, SE=Standard error

TP and albumin concentration increased significantly (p<0.01) from 0 to 14 days as well as on 45^th^ day post-partum. Globulin increase was non-significant during the study. Similar values were observed between control and treatment groups on 30^th^ and 45^th^ day post-partum for TP and 21^st^ day and 45^th^ day post-partum for albumin. TG values were significantly (p<0.01) different from 0 to 30^th^ day post-partum, and its highest and lowest values were observed on 0 day and 45^th^ day, respectively. NEFA values were significant (p<0.01) during the post-partum period. NEFA concentration was significantly (p<0.01) higher in the control group as compared to treatment group where it showed a decreasing trend from 0 to 45^th^ day post-partum. Glucose concentration decreased from the day of kidding until 14^th^ day post-partum followed by an increased up to 45^th^ day post-partum. As compared to treatment group significant (p<0. 01) differences in glucose values were seen in control group wherein it was lower on the day of kidding and higher on the 7^th^ and 14^th^ day. Urea concentration was lowest on the day of kidding to highest on 45^th^ day post-partum. Values of urea concentration on the 14^th^ day of post-partum were similar to values in the control group.

The values of some biochemical parameters in both the group (especially on some post-partum days of treatment group) were slightly lower or higher than that of the normal range. The variation could be due to stress on the day of kidding and during the post-partum period.

## Discussion

A significantly lower Hb concentration was observed on the day of kidding which subsequently increased significantly up to 45^th^ day of lactation. Increase in Hb during the post-partum period may be due to higher demand of oxygen and requirement of higher metabolic rate [4]. Increase in Hb during the post-partum period has been reported [5]. Non-significant difference on 0 day and 7 days after kidding have been reported by Rejitha and Karthiayini [6] in Malabari goats, −3 to +3 weeks of kidding by Tharwat *et al.*, [7].

Increasing trend in PCV was observed from 0 to 45 days post-partum. Decrease in PCV on the day of parturition has also been reported by Tharwat *et al.*, [7]. The decrease in PCV on the day of parturition has been associated with stress related to parturition [8]. The decrease in PCV on the day of parturition may be attributed to hemodilution effect resulting from an increase in plasma volume or increased water mobilization to mammary gland through the vascular system [9]. However decreases in PCV up to 3^rd^ week of post-partum have been observed by Iriadam [10], while decrease in PCV during both late pregnancy and early lactation has also been reported by Araz [11].

The present study revealed non-significant difference during post-partum period in TEC that are in agreement to that reported by Rejitha and Karthiayini [6]. In general, red cell parameters decrease during gestation and remain low for a few weeks post-partum as has been observed in cows, mares, sows, ewes, and bitches [12]. However, a great variation in hematological parameters exists between various breeds of goat [13].

Significantly (p<0.01) low value of TLC was observed on 0 day and values on 7^th^ to 45^th^ day post-partum differed non-significantly. Lowest TLC was found on the day of kidding that may be due to low immunity or stress. A significant difference in lymphocyte, MID % (mid-sized cells), and granulocyte % was observed on the 0-45 days in the present study. The present finding of increase in granulocyte on the day of kidding are similar to the findings of Iriadam [10]. Granulocyte to lymphocyte ratio increase during the post-partum period which may be attributed to stress that stimulates secretion of adrenocorticotropic hormone which in turn induces the adrenal cortex to produce glucocorticoids, involved in the mobilization of granulocytes from body pool into the peripheral circulation [14]. In the present study also the ratio of granulocyte: Lymphocyte was highest on the day of kidding.

TP and albumin concentration increased significantly (p<0.01) from 0 to 14 days as well as on 45^th^ day post-partum while the difference was non-significant for globulin. TP increased during the post-partum period that may be because of increase in globulin resulting from the formation of immunoglobulin. The finding of the present study of the increase in TP and globulin in post-partum are similar to that of Tharwat *et al.*, [7]. Increase in TP but non-significant decrease in albumin in 3^rd^ week after parturition has also been reported by Iriadam [10]. Increase in albumin in 1^st^ week followed by decrease in 2^nd^ week post-partum has been reported during post-partum Iriadam [10].

A significant (p<0.01) difference was observed from 0 to 30^th^ day post-partum in the levels of TG. High TG concentration during the 1^st^ week before parturition followed by a significant decrease on 2 days post-partum has been reported by Skotnicka *et al.*, [15]. The onset of lactation has significant effect on TG as well as cholesterol concentration. During the lactation period, lipogenesis and esterification are reduced, and free fatty acid mobilization is stimulated by an increase in nor-epinephrine and epinephrine secretion. The activity of lipoprotein lipase is increased in mammary gland and decreased in adipose tissue. However, contradictory results of lowest TG on the day of kidding as compared to present findings were reported by Sadjadian *et al.*, [16]. Decrease in TG concentration during the lactation period may be due to catabolism of TG for the supply of energy for milk synthesis.

NEFA values were significantly different (p<0.01) during the post-partum period. Decreasing trend was observed from 0 to 45^th^ day post-partum. Peak level of NEFA concentration on the day of kidding followed by decrease up to 45^th^ days are in agreement to that of Sadjadian *et al.*, [16] in Saanen does. The increase in NEFA concentration at the time of parturition may be due to high energy requirement for parturition. Increase in plasma lipolytic hormones prior to parturition may be contributing to increasing plasma NEFA concentration. Increase in NEFA indicates a deficit in energy intake due to the mobilization of fat and increase in free fatty acid. The decrease in NEFA concentration was found to be significantly decreasing during different weeks. Elevated plasma concentration of NEFA occurs simultaneous to increased rate of lipolysis in adipose tissue and has been reported in goats during late pregnancy and early lactation [17]. Blood concentration of NEFA is linked to energy balance and 0.20-0.21 mmol/L NEFA concentration has been suggested for lactating does at zero energy balance [18]. Plasma concentrations of NEFA may be possible diagnostic markers of impaired immunity and a higher risk of infections around parturition [19].

Peak level of glucose on the day of kidding was followed by a decrease during 2^nd^ week post-partum. Similar findings are reported by Sadjadian *et al.*, [16]. Increase in glucose concentration on the day of parturition may be due to metabolic changes toward gluconeogenesis [20] and hormonal changes at parturition that promote gluconeogenesis and glycogenolysis [21]. High level of glucose on the day of kidding indicates animals were in positive energy balance. The calorie protein ratio was higher on 0 day as compared to 7^th^ and 14^th^ day. Increase in glucose may also be due to lower concentration of insulin. Decreasing blood glucose concentration during first 2 weeks of lactation appears to be related to high energy demand especially in high milk producing breeds of goats and increasing glucose concentration after the 2^nd^ week of lactation may be due to the recovery of feed intake and decreasing negative energy balance. At the time of parturition, glucocorticoids and estradiol concentration reaches the peak level and even the insulin response. A significant increase in glucocorticoid causes liver glycogenolysis and mobilization of amino acid for gluconeogenesis. The decrease blood glucose with the advancement of days of lactation may be attributed to the synthesis of lactose with increased milk production [21].

Urea concentration increased significantly (p<0.01) from 0 to 45^th^ day post-partum. High level of blood urea nitrogen (BUN) on the 21^st^ day post-partum and lowest level on the day of kidding has been reported [16]. The decrease in serum BUN around parturition may be associated with the decline in feed intake due to stress and hormonal changes during the kidding. Similar findings of a significant increase in BUN during the post-partum period have been reported in different goat breeds [16].

## Conclusion

Hematobiochemical parameters are indicative of health status of Surti goats. Even though hematological parameters measured were in normal range, significantly low values of Hb, PCV, and TLC during first 2 weeks as compared to the control group are indicative of stress. Parturition and post-partum stress led to wider variations in biochemical parameters as compared to normal range. TP and urea increased parallel to one another during the post-partum period. Catabolism of protein takes place during the first 2 weeks post-partum. Decrease TGs and increase in NEFA during the transition period are suggestive of utilization of lipids for the supply of energy. High glucose level on 0 day indicates that the animals were in positive energy status.

## Authors’ Contributions

SSC designed and supervised the experiment. TDM conducted the experiment and also with the help of VKS and SBP conducted the laboratory analysis of the samples. TDM along with SSC analyzed the data and prepared the manuscript. SSC, GP, VKS, SBP, and TDM reviewed the manuscript. All authors read and approved the final manuscript.
